# The case for excision and primary anastomotic urethroplasty for bulbar urethral stricture

**DOI:** 10.1590/S1677-5538.IBJU.2016.05.02

**Published:** 2016

**Affiliations:** 1 Department of Urology, University of Texas Southwestern Medical Center, Dallas, TX, USA

With an estimated prevalence of 296 to 627 per 100.000 men, male urethral stricture disease imposes a significant burden on the health care system (1, 2). Urethroplasty has demonstrated durable, high success rates in the management of a wide spectrum of stricture disease, far exceeding that of the more commonly performed but less successful direct vision internal urethrotomy (DVIU) (1, 3-5). While procedure selection depends on stricture characteristics (length, location, and etiology), the high success rate of excision and primary anastomotic (EPA) urethroplasty makes it the procedure of choice for most strictures of the bulbar urethra (6). However, concerns regarding the effect of urethral transection on male sexual health has led some centers to advocate for substitution urethroplasty (7), likely contributing to an increase in these procedures (8). Our objective is to review the literature supporting EPA urethroplasty in strictures of the bulbar urethra.

## Success of EPA urethroplasty

A 25-year meta-analysis of the contemporary EPA urethroplasty literature demonstrates a uniformly high level of success (>90%) for EPA of bulbar strictures (6), superior to the results of any other method of bulbar urethroplasty (9, 10). In fact, a 4-fold increase in stricture recurrence has been reported in substitution urethroplasty when compared with EPA (11). While attempts to demonstrate equivalency between substitution urethroplasty and EPA have been reported, these studies suffer from patency rates that fall short of that ordinarily reported for EPA, as well as discrepant follow-up between treatment groups (12).

Unfortunately, DVIU remains the most frequently used treatment for anterior urethral stricture, despite its inferior results compared to urethroplasty (1, 3, 13). This is often attributed to the simplicity and speed of endoscopic treatment and a lack of urologists trained in urethroplasty techniques, especially in various rural geographic locations (2, 14). At best, initial DVIU for short, non-traumatic strictures of the bulbar urethra has been associated with a success rate of only 39-73%, with subsequent attempts performing even worse (4, 5, 15). It is clear that after prior failed urethrotomy the complexity of the stricture increases (16). Current recommendations allow for a single attempt at DVIU in a well-selected patient before referring stricture patients for definitive urethroplasty (17, 18).

## UTSW EPA experience

Our EPA technique emphasizes complete scar excision, while maintaining the vascular integrity of the spongiosum. Though EPA failure is rare, it is our belief that many failures are caused by inadequate proximal urethral dissection, leading to incomplete resection of diseased tissue. In order to optimize the proximal dissection, we utilize antegrade instrumentation via a suprapubic tract (if present), or retrograde placement of a guidewire, which aides in the identification of the proximal lumen. With control and adequate visualization of the proximal urethral stump, the strictured mucosa and associated spongiofibrosis is completely excised, leaving well-vascularized urethral tissue for a healthy anastomosis. We advocate performing a two-layer ventral anastomosis after urethral transection for precise re-approximation of the robust vasculature of the ventral spongiosum. We avoid additional maneuvers such as corporal splitting whenever possible, as they may further compromise antegrade and retrograde corporal blood flow to the urethra. Instead, we facilitate urethral lengthening by aggressively mobilizing the urethra from its ventral crotal attachments while preserving dorsal perforating vasculature as much as possible. Our results underscore the value of incorporating these various alternative technical measures to avoid urethral ischemia.

Adequate urethral length after transection is essential for a tension-free anastomotic repair. Despite traditional recommendations that the EPA technique be limited to strictures 2cm or less long (21), our experience suggests that this underestimates the potential urethral length that can be mobilized via a perineal dissection (22). Male urethra has been shown to be exceptionally extensible, with a possible additional 65% of length obtained after mobilization, allowing for a tension-free anastomosis even in longer strictures (23). It has been our experience that up to 5cm resection is possible in the proximal bulb (lower half of perineal incision) in selected favorable cases, a 2cm limit is more customary in the distal bulb (upper half of perineal incision).

## Complications of EPA urethroplasty

Contemporary analysis of urethroplasty in the United States reveals a low post-operative complication rate of 6.6% (8). Compared to anastomotic urethroplasty, substitution urethroplasty is associated with a 5-fold increase in complications, though direct comparisons of the complication profiles are scant (11). It is clear however, that anastomotic repairs obviate the time and trauma needed for harvesting of flap or graft, thus avoiding those inherent complications (24, 25).

The complication of erectile dysfunction after anterior urethroplasty is a controversial topic with many divergent opinions (7). Overall, urethroplasty has been shown to cause transient post-operative erectile functional decline in up to 40% of patients (26). Multiple risk factors appear to be involved, such as patient age, stricture length and location, and type of reconstruction (26-28). While ED in anastomotic repairs has been shown to be significantly higher at three months post-operatively (26, 28, 29), there is no demonstrable difference at 6 months and beyond (26, 28, 30-35). In fact, overall sexual dysfunction after urethroplasty lasting beyond 6 months has been shown to be on par with circumcision (27).

## CONCLUSIONS

Excision and primary anastomotic urethroplasty demonstrates the highest long-term success rate among available techniques for bulbar urethral strictures. The overall complications of EPA are low, and erectile dysfunction, if encountered, generally resolves after six months of recovery. EPA should be considered the gold standard in treatment of bulbar strictures.
